# Plant nutrient removal and soil residual chemical properties as impacted by maize planting date and density

**DOI:** 10.1371/journal.pone.0299193

**Published:** 2024-03-28

**Authors:** Koffi Djaman, Dorlote S. Djaman, Naveen Puppala, Murali Darapuneni

**Affiliations:** 1 Department of Plant and Environmental Sciences, Agricultural Science Center at Farmington, New Mexico State University, Farmington, NM, United States of America; 2 Faculté des Sciences de l’Agriculture l’alimentation, Université Laval, Pavillon Paul*-*Comtois 2425, Québec, Canada; 3 Department of Plant and Environmental Sciences, Agricultural Science Center at Clovis, New Mexico State University, Clovis, NM, United States of America; 4 Department of Plant and Environmental Sciences, Agricultural Science Center at Tucumcari, New Mexico State University, Tucumcari, NM, United States of America; Central South University of Forestry and Technology, CHINA

## Abstract

This study aimed to measure maize (*Zea mays*) plant nutrient content and nutrient removal in grain, and to evaluate the residual soil nitrogen, phosphorus, and potassium as impacted by planting date and density. Field experiments were conducted to evaluate six plant densities and seven planting dates using a split-split plot design with three replications. Besides the crop growth and yield parameters, six plants were collected at the maturity and soil was sampled from each plot for nutrient analysis. Plant N, P, and K concentrations varied with planting date and density and within the ranges of 0.6–1.024%, 0.054–0.127%, and 0.75–1.71%, respectively. Grain N, P, and K concentrations decreased with plant density and varied from 1.059 to 1.558%, 0.20 to 0.319%, and 0.29 to 0.43%, respectively. Soil residual nutrient varied with depth, planting density and date. Residual N concentration in the topsoil varied from 0.6 to 37.2 mg kg^-1^ in 2019 and from 1.5 to 11.2 mg kg^-1^ in 2020 and was high under the last two planting dates. Soil residual N concentration was higher in the second layer than in the topsoil. The N concentration in the third layer varied from 0.1 to 33.2 mg kg^-1^ and was impacted by plant density. Topsoil P did not vary among planting dates and densities. The second and third soil layers P concentration was not affected. There was 83% increase in topsoil K in 2020 compared to 2019, and a decrease of 65 and 23% in soil K was observed in the second and third soil layers, respectively. For maize production system sustainability, future research should use a holistic approach investigating the impact of planting date, plant density on crop growth, yield, nutrient uptake and remobilization, and soil properties under different fertilizer rates to develop the fertilizer recommendation for maize while reducing the environmental impact of the production system.

## 1. Introduction

Most of crop fertilizer recommendations are based on the soil residual nutrient concentrations, crop species, hybrids, or varieties. To our knowledge, crop fertilizer recommendations are not related to plant densities. Optimization strategies for maize production have been targeting planting density and planting date for improving crop yield and farm profit. Several studies have reported different plant densities that maximize maize grain yield [1–6]. Different planting dates have been tested for maize’s high yield and grain quality [6–18]. From other studies combining planting date and plant density, it was concluded that the choice of maize plant densities should be based on seasonal weather forecast to maximize opportunities for higher yields [6,19].

Three major macronutrients such nitrogen (N), phosphorus (P) and potassium (K) are very important in crop production and should be well managed at field, watershed, and regional level for productions economics, system sustainability, and conservation. Across the U.S., maize fertilizer recommendations are based on the expected yield objectives and the soil residual nutrients [20]. Optimizing maize plant density and planting window targets maximum grain yield and different resources use efficiencies such as nitrogen use efficiency, water use efficiency, and radiation use efficiency [5,6,21–23].

Nutrient concentrations in maize plants are generally dependent on plant density. Lower planting densities tend to show higher nutrient concentrations mostly due to a luxurious uptake and the optimum densities nitrogen is effectively used [21,24,25]. Raymond et al. [21] reported variation in nutrient uptake by maize with the highest under the highest plant density. Yan et al. [26] found low nitrogen uptake under high plant densities of maize with crowding stress reduces the ability of plants during the post-silking period. Feyissa et al. [27] highlighted the effectiveness of the combined strategy of optimized plant density, balancing NPK input, and innovative NPK fertilizer on sustainable maize production. Ciampitti et al. [25] reported that maize cumulative P uptake was significantly influenced by planting density and nitrogen fertilizer rate from early vegetative to silk emergence, after which the planting density effect disappeared while the Potassium uptake was significantly affected by planting nitrogen fertilizer rate during the entire season. Lower plant N uptake was observed in the high planting density [28]. Ottman and Welch [29] found a non-consistent effect of planting patterns on nutrient concentration in maize grain, stover, or cob.

Plant canopy and microenvironment are modified by plant density, which affects the capability of plants to acquire mineral nutrients from the immediate rhizosphere due to competition among plants for space, light, and nutrients [30,31]. The concentration of macronutrients (N, P, K) in many crops is known to decrease with the increase in plant biomass [20,25,32–34]. Zohaib et al. [35] found a decreasing trend in cotton seed nutrient concentration at higher plant densities. Very limited information is available on the residual soil nutrient concentrations under different maize plant densities. In general, 90% of maize plant roots are located within the 20 cm topsoil layer with 60% within the 10 cm radius from each plant [36]. Nutritional gradient zone around each individual dictates mineral nutrient absorption by roots of a nutritional gradient zone around each individual. With overlapping nutritional gradient zones of neighboring plants under high plant densities, nutrient concentration in the overlapped area tremendously decreases because of interactions between adjacent roots, resulting in reduced root absorption efficiency [37].

Plant nutrition and soil chemical composition are usually acted by plant density and planting date under a temperate climate. Proper planting density improves the plant-soil environment [38] while the higher densities increase competition among plants for the soil nutrient uptake with a significant decrease in soil N, P, and K contents [39–41]. Postma et al. [42] reported up to 2 m soil profile nitrogen depletion under high plant densities compared to low plant densities with a greater deeper down, in the 1–2 m soil layer, than in the 0–1 m soil layer. Duan et al. [43] pointed out that excessively high plant density is not beneficial to the long-term maintenance of soil fertility in Chinese fir plantations while soil N and P are depleted under high plant densities. At a high plant density of maize, there is an intensified root competition for soil nutrients [41,44]. Increasing plant density accelerates soil nutrient depletion however, the increase in maize above biomass may contribute to nutrient partially relocated from the deep soil layers to the surface soil layer with biomass incorporated to the soil during tillage after harvest [45]. Chen et al. [46] indicated that integrated soil–crop management systems represent a priority for agricultural research and implementation under cereal grains production to overcome the dual challenge of substantially increasing yields of cereal grains while at the same time reducing the very substantial environmental impacts of intensive agriculture.

The objectives of the present study were to: i) measure grain maize N, P, and K uptake under different plant densities and planting dates, (ii) quantify the impact of different planting density and planting dates on residual soil N, P, K under sprinkler irrigation in northwest New Mexico climate and typical sandy loam soil conditions.

## 2. Materials and methods

### 2.1 Site description, experimental design, and general soil and crop management practices

**Fig 1:**
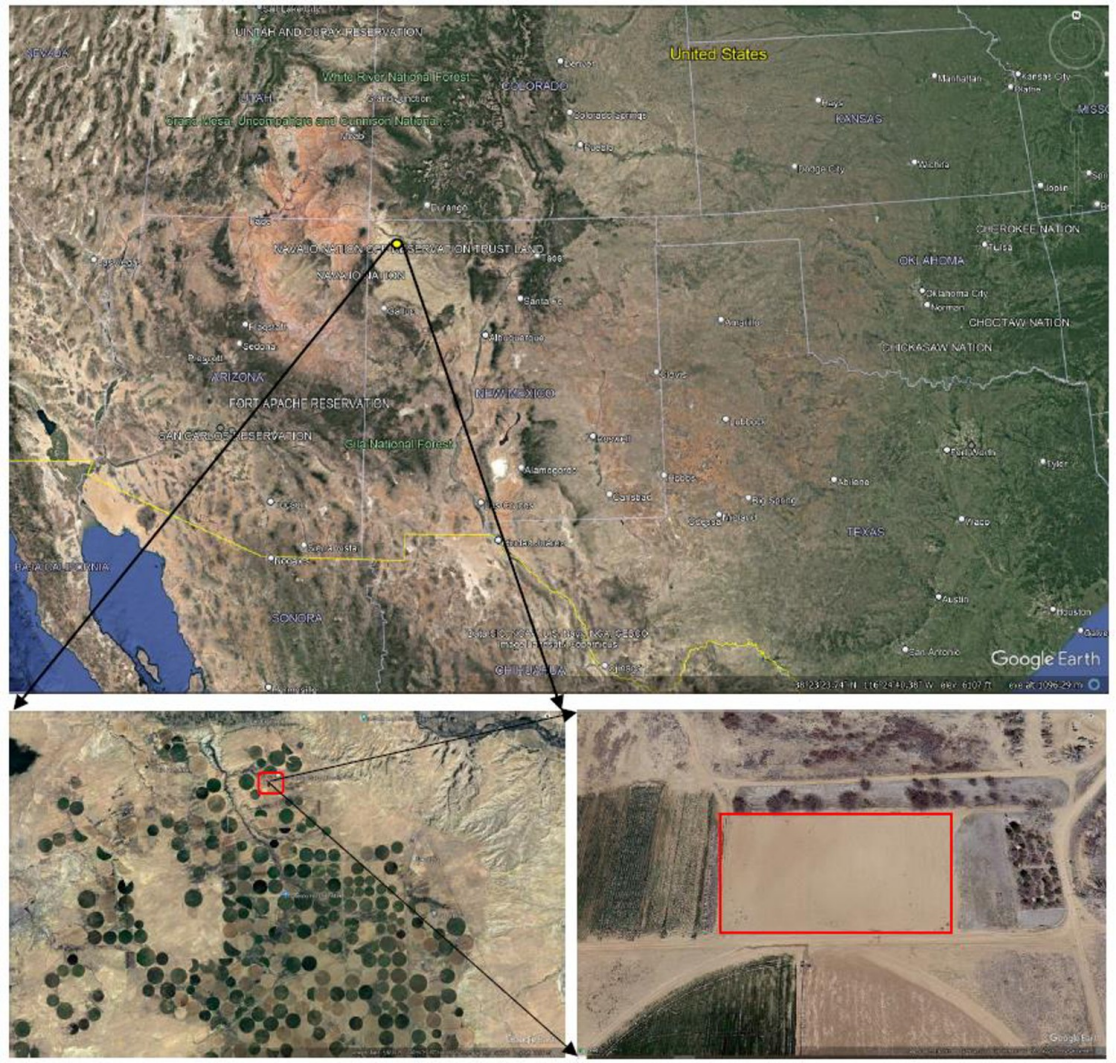
Presentation of the study site in San Juan County, Northwestern New Mexico (yellow dot on the US map) and the NMSU Agricultural Science Center at Farmington (red square on the lower left map). The green/yellow circles are the center pivot irrigated fields, the red rectangle on the lower right map is the actual plot used for the present research (Image Date4/6/2019; 36o41’12.06” N 108o18’40.32” W elev. 5636 ft). (downloaded from Google earth on December 30, 2023).

This study was conducted at the Agricultural Science Center at Farmington, New Mexico (latitude 36.69′ North, longitude 108.31′ West, elevation 1720 m) for the 2019 and 2020 growing seasons (Fig 1). The experiment site is characterized by a semiarid climate with a long-term average annual precipitation of 216 mm and the maize growing season precipitation average of 101 mm [47,48]. The long-term average annual maximum and minimum temperatures are 17.2°C and 2.2°C, respectively while the typical growing season average annual maximum and minimum temperatures are 20°C and 10.8°C. Details of the 2019 and 2020 weather conditions can be found in Djaman et al. [6]. The soil at the experimental site is a Doak sandy loam with a pH of 8.2 and low organic matter content. Hybrid Maize DKC53-45RIB was planted at weekly interval under six plant densities (54700, 64600, 74600, 88000, 101700, and 120100 plants ha^-1^) and seven planting dates in 2019 (April 23, May 1, May 7, May 14, May 22, May 30, and June 5, named D1, D2, D3, D4, D5, D6, and D7, respectively) and in 2020 (April 21, April 30, May 7, May 18, May 27, June 3, and June 10, named D1, D2, D3, D4, D5, D6, and D7, respectively). The experiment was set up under a split-split plot design with three replications. Maize planting dates were attributed to the main plots and plant densities were attributed to the subplots. An experimental unit size was 9.14 m over 6.10 m, and it comprised eight rows of maize plants with 0.76 m of row spacing. Plots were sprinkler irrigated based on maize actual evapotranspiration. Before planting, 56 kg ha^-1^ of the mix of mono-ammonium phosphate (11-52-0), potassium chloride (0-0-60) and urea (46-0-0) was applied, and liquid urea (32-0-0) was applied throughout maize vegetative and reproductive phases by fertigation at a total applied rate of 140 kg N ha^-1^. Plots were kept weed-free using glyphosate and hand weeding if necessary. All plots were managed uniformly under different densities and planting dates. At harvest, three middle maize rows of an experimental unit were combine-harvested, and the plot yield adjusted to 14% moisture content was reported in Mg ha^-1^. More details about the experiment can be found in Djaman et al. [6].

### 2.2 Plant and soil sampling

Before planting, soil samples were taken from the 0 to 90 cm soil depth at 30 cm increments for N, P, and K analysis. Soil samples were also taken within each experiment unit at the end of each growing season. Soil samples were collected on seven sampling dates. The soil was sampled at mid-distance between two plants in a crop row. Three cores were mixed per depth and per treatment and sent to the soil processing laboratory to determine the concentrations of P, K, and NO^3^–N for each soil depth. At crop physiological maturity, six plants from all three replications of each treatment were selected randomly to determine the aboveground biomass production and the chemical analysis. Plant biomass samples were not taken in 2020 because of a big wind at plant full maturity with dry leaves and that wiped off plant leaves and taking the samples without leaves may introduce bias in the plant N, P, and K analysis data. Plant and grain samples were weighed and conserved in a freezer until being sent to the Ward Laboratory (https://www.wardlab.com/) for N, P, and K analysis. The plant, grain, and soil were analysis for NPK content according to the procedure described by Ward [49]. Biomass and grain nutrient concentrations are expressed as a percentage on a dry-weight basis, and the soil N, P, and K concentrations are expressed in mg kg^-1^. Nutrient (N, P, and K) uptake in grain was calculated by multiplying the grain yield by the grain nutrient concentration. Total plant nutrient uptake was calculated by multiplying the plant nutrient concentration by biomass production.

### 2.3 Statistical analysis

Maize grain yield, soil nutrient content, and grain and maize stover nutrient content data were analyzed using CoStat statistical software [50]. Following the analysis of variance procedures, differences among treatment means were determined using the least significant difference (LSD) comparison method. Means significance was analyzed at the 95% confidence level. The relationships between maize grain N, P and K removal and plant densities were fitted to the second order polynomial.

## 3. Results

### 3.1 Plant nutrient concentration as function of plant density and planting date

Maize planting date showed highly significant effect on plant N, P, K content at plant maturity while the planting density showed non-significant effect on plant N, P, K content (Table 1). Both factors in addition to the planting year showed significant effect on grain N, P, K contents. Grain N, P, K contents were also impacted by only the interaction Planting date and the year of the experiment.

**Table 1:** Summary of the analysis of variance (ANOVA) of the effect of maize planting date and density on maize biomass and grain N, P, K contents1.

Plant material	Source	df	Nitrogen	Phosphorus	Potassium	Comments
			p-value	p-value	p-value	
Plant biomass	**Main Effects**					
	Planting Date	6	0.0112	0.0036	0.0000	Significant effect on plant N, P and K
	Planting Density	5	0.0508	0.2783	0.5144	Non-significant effect on plant N, P and K
	**Errors**	30				
Grain	**Main Effects**					
	Planting Date	6	0.0000	0.0083	0.0034	Significant effect on grain N, P and K
	Planting Density	5	0.0000	0.0000	0.0019	Significant effect on grain N, P and K
	Year	1	0.0000	0.0000	0.0271	Significant effect on grain N, P and K
	**Interactions**					
	Planting Date * Planting Density	30	0.8497	0.8139	0.8458	Non-significant effect on grain N, P and K
	Planting Date * Year	6	0.0002	0.0058	0.0000	Significant effect on grain N, P and K
	Planting Density * Year	5	0.9119	0.8890	0.6199	Non-significant effect on grain N, P and K
	**Errors**	30				

**Fig 2:**
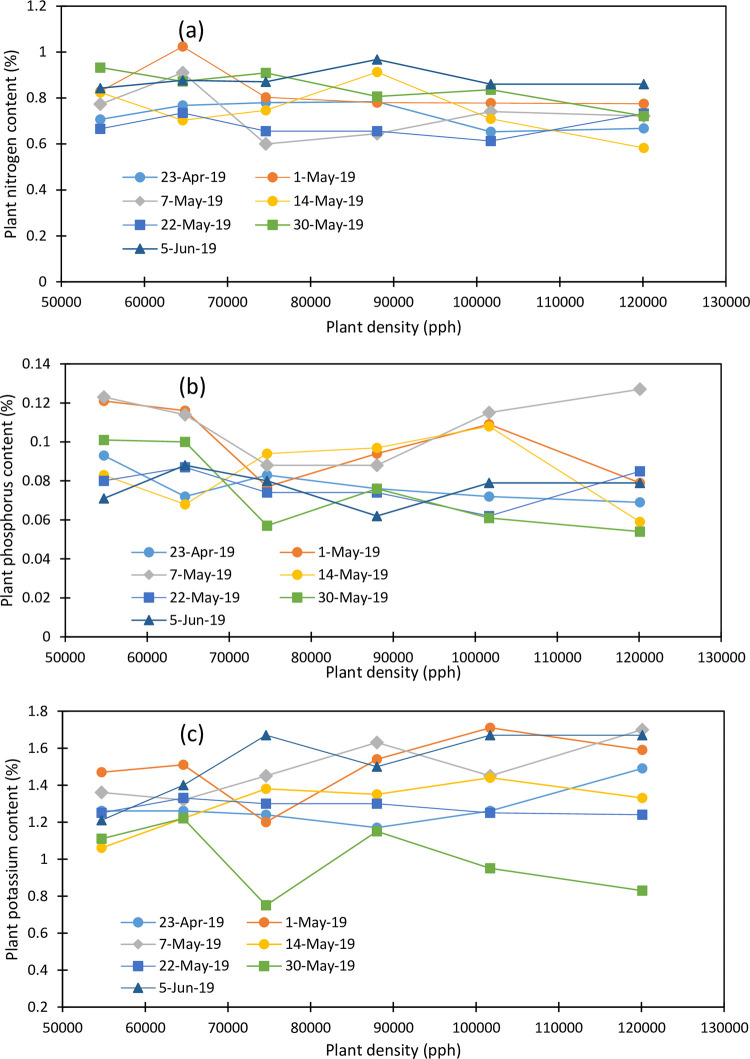
Variation in plant (a) nitrogen, (b) phosphorus and (c) potassium as function of planting date, and plant density (2019).

The trend in plant nutrient concentrations is shown in Fig 1. While there was no clear variation in plant nitrogen, phosphorus, and potassium concentrations as a function of plant density, the late plantings showed higher nutrient concentrations in maize plants compared to the early plantings. Maize plant nitrogen concentration varied from 0.6 to 1.024% and averaged 0.80, 0.84, 0.77, 0.79, 0.74, and 0.72% under 54700, 64600, 74600, 88000, 101700, and 120100 plants per hectare (pph), respectively (Fig 2A). The lowest plant N concentration occurred under the highest plant density and the highest plant N concentration occurred under the 64600 pph. The Maize plant N concentration was also impacted by the planting date and averaged 0.73, 0.83, 0.73, 0.75, 0.68, 0.85, and 0.88% for the April 23, May 1, May 7, May 14, May 22, May 30, and June 5 plantings, respectively. The last two planting dates obtained the highest plant N concentration. This might have been the shortness of N translocation from the leaves to the plant reproductive parts before plant senescence and the impact of the first fall frost. The May 22 planting obtained the lowest N concentration in the maize plant.

Maize plant phosphorus concentration varied with plant density and planting date and ranged between 0.054 and 0.127% (Fig 2B). Overall, the maize plant showed a slightly decreasing trend with increasing plant density from 0.096 to 0.079% while it increased from 0.078% for April 23 to 0.109% for the May 7 planting and decreased thereafter to 0.075% for the last plantings. The plant density of 54,700 pph obtained the highest plant phosphorus concentration and the 120,100 pph obtained the lowest plant phosphorus concentration.

Maize plant potassium concentration was discarded with plant density and planting date and varied from 0.75 to 1.71% (Fig 2C). Maize plants showed an increasing trend in plant potassium concentration with plant density. However, plant K concentration dropped at 74600 pph and increased as plant density increased. Maize K concentration increased from the April 23 planting to May 1 planting. Plant K concentration decreased with delayed planting to the minimum of 1.002% for the May 30 planting and increased to 1.52% for the June 5 planting. Overall, the May 1 and June 5 plantings showed the highest and similar plant K concentration while the May 30 planting showed the lowest plant K concentration.

### 3.2 Grain nutrient concentration as function of plant density and planting date

**Fig 3:**
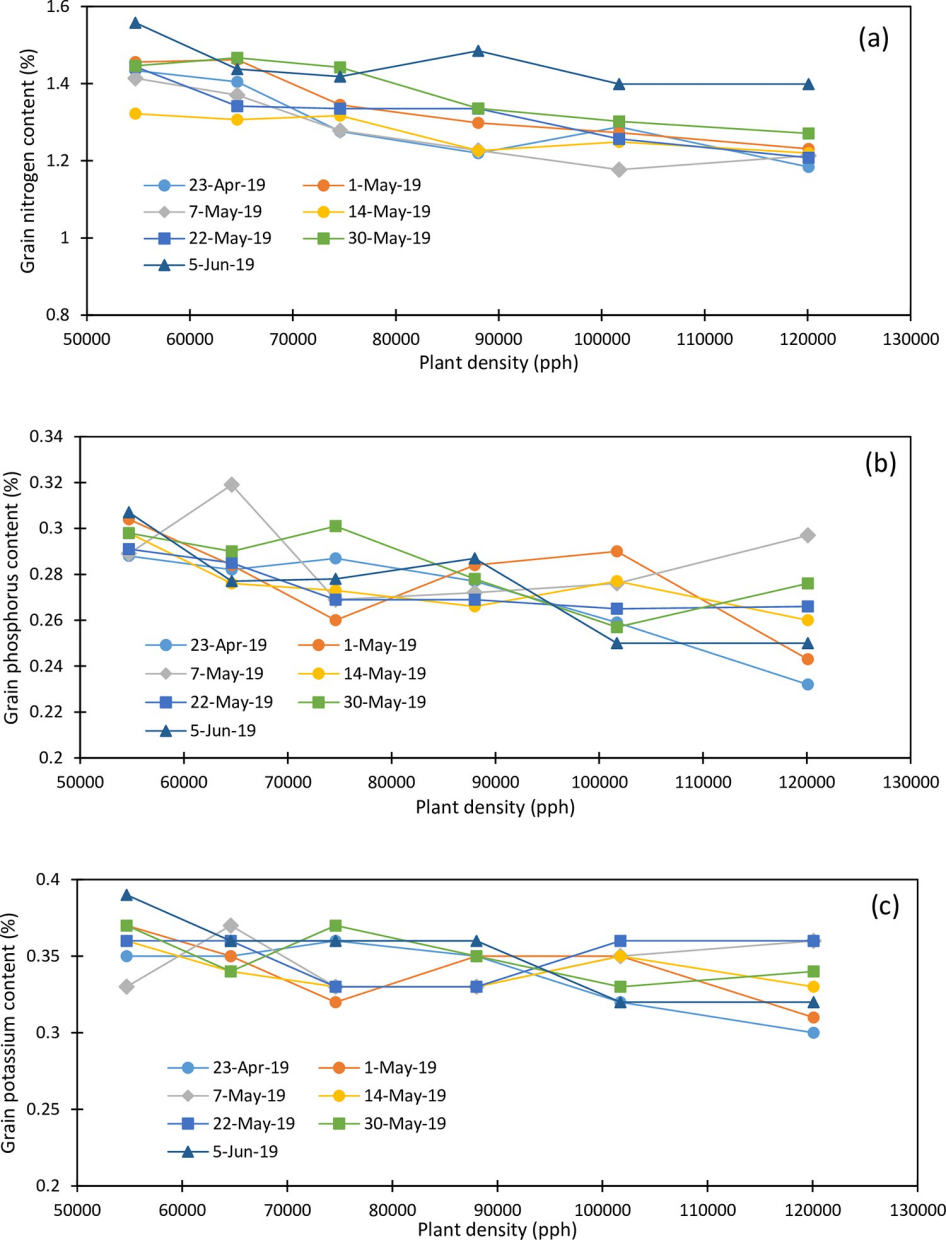
Variation in grain (a) nitrogen, (b) phosphorus and (c) potassium as function of planting date, and plant density (2019).

**Fig 4:**
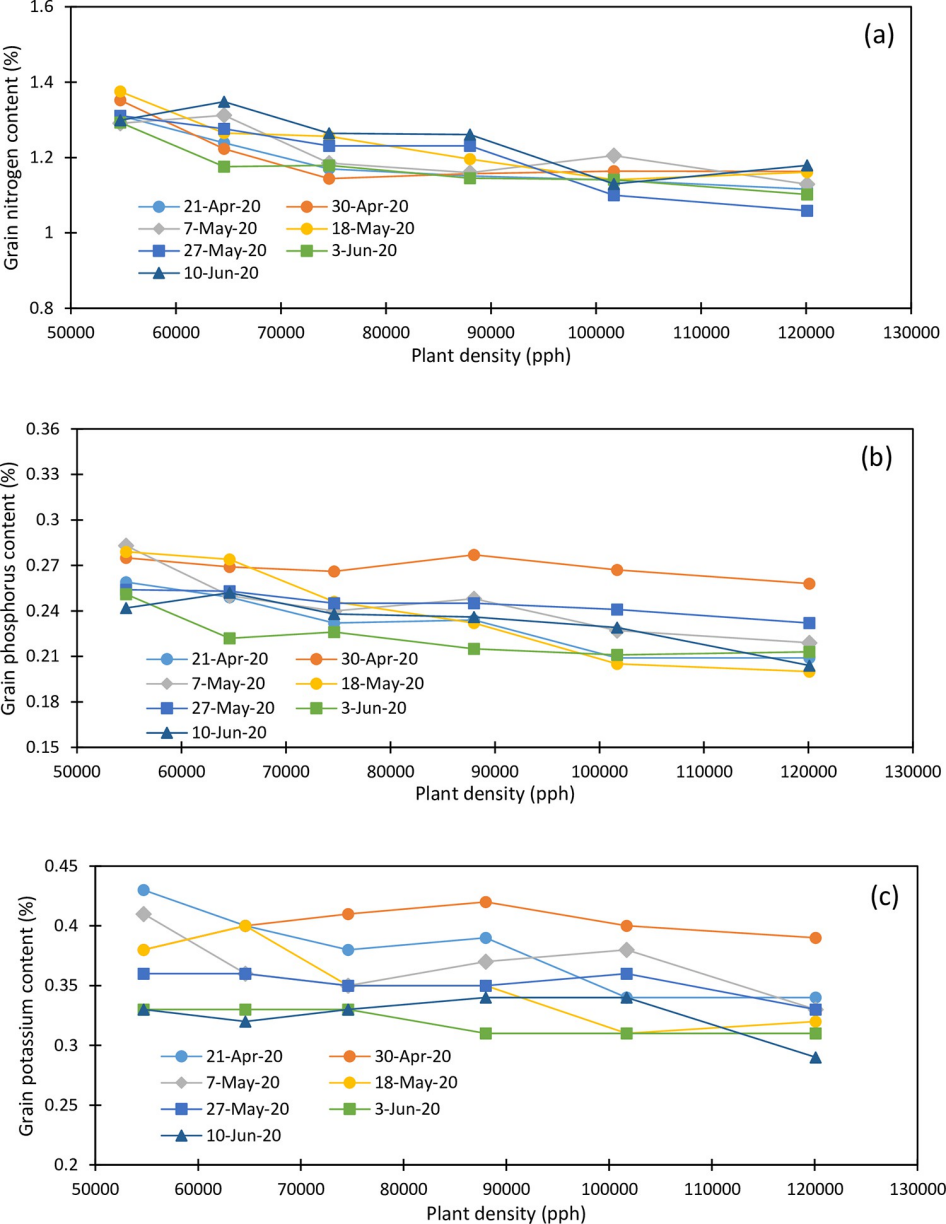
Variation in grain (a) nitrogen, (b) phosphorus and (c) potassium as function of planting date, and plant density (2020).

Maize grain yields for the 2019 and 2020 growing seasons are presented in Table 2. Maize grain N concentration varied from 1.177 to 1.558% in 2019 (Fig 3A) and from 1.059 to 1.375% in 2020 (Fig 4A). Grain N concentration decreased linearly with plant density and averaged 1.439, 1.399, 1.344, 1.304, 1.278, and 1.247% under 54700, 64600, 74600, 88000, 101700, and 120100 pph in 2019. Grain N concentration decreased linearly with plant density and averaged 1.319, 1.263, 1.204, 1.186, 1.146, and 1.123% under the respective plant densities in 2020. Grain N concentration increased from April 23 planting (1.301%) to May 1 planting (1.344%) and decreased to 1.280% at the May 7 planting and 1.274% at the May 14 planting and it linearly increased to 1.450% at the June 5 planting in 2019. In 2020, grain N concentration increased with the planting date from April 21 (1.188%) to May 18 planting (1.232%) and decreased to 1.201% and 1.173% for the May 27 and June 3 plantings, respectively, and increased to 1.2475 for the June 10 planting in 2020. During both study growing seasons, the lowest plant density obtained the highest grain N concentration, and the last planting date always showed the highest grain N concentration. The May 14 planting showed the lowest grain N concentration in 2019 while it was obtained under the June 3 planting in 2020.

**Table 2:** Maize grain yield (Mg ha^-1^) as function of planting date and plant density.

Plant density	Planting dates (2019)						
	23-Apr	1-May	7-May	14-May	22-May	30-May	5-Jun
54,700	12.93 a	12.23 a	12.89 a	14.06 a	11.63 a	11.84 a	10.89 a
64,600	13.14 a	13.63 b	13.68 b	14.48 b	13.01 b	13.48 b	12.48 b
74,600	14.96 b	14.21 c	14.53 c	14.92 c	14.35 c	14.69 c	13.6 d
88,000	16.65 d	16.22 d	16.37 d	15.27 d	14.49 c	15.55 d	13.93 de
101,700	16.49 cd	16.78 e	16.29 d	14.99 c	15.47 e	16.77 e	13.45 d
120,100	16.25 c	15.98 d	14.62 c	14.49 b	14.96 d	16.54 e	12.87 c
Plant density	Planting dates (2020)						
	21-Apr	30-Apr	7-May	18-May	27-May	3-Jun	10-Jun
54,700	11.87 a	13.41 a	12.91 a	13.35 a	12.97 a	12.78 a	13.18 a
64,600	13.42 b	13.65 b	14.23 b	15.09 b	14.47 b	13.38 b	14.70 c
74,600	14.03 c	14.17 c	14.71 c	16.24 c	14.84 c	14.61 c	14.91 c
88,000	15.52 e	15.36 d	17.00 e	17.77 e	16.12 e	16.01 e	16.03 e
101,700	14.89 d	13.89 b	16.60 d	17.34 d	15.10 cd	15.48 d	15.37 cd
120,100	14.71 d	12.98 a	14.91 c	17.46 d	14.28 b	13.61 b	13.67 b

*Numbers followed by different letters are significantly different.

Grain P concentration varied from 0.232 to 0.319% in 2019 (Fig 3B) and from 0.20 to 0.283% in 2020 (Fig 4B) and averaged 0.296, 0.288, 0.277, 0.276, 0.268, and 0.261% under 54700, 64600, 74600, 88000, 101700, and 120100 pph, respectively, in 2019 and 0263, 0.253, 0.242, 0.241, 0.227 and 0.219% in 2020 under the respective plant densities. Plant densities of 74,600 and 88,000 pph showed similar P concentrations of about 0.277% in 2019 and 0.242% in 2020. Grain P concentration was also impacted by the planting date. It increased from April 23 planting (0.271%) to May 7 planting (0.287%) and decreased under the May 14 and May 22 plantings (0.275 and 0.274%, respectively), increased under the May 30 planting (0.283%) and dropped at the June 3 planting (0.275%) in 2019. In 2020, grain P concentration increased slightly from the April 21 planting (0.232%) to the April 30 planting (0.269%) and slightly decreased with the delayed planting. The last planting showed an increased grain P concentration compared to the previous plantings as shown in 2019. Overall, the May 7 planting obtained the highest grain P concentration of 0.287% in 2019 and the April 30 planting showed the highest grain P concentration of 0.268% in 2020 while the April 23 planting in 2019 and June 3 planting in 2020 showed the lowest grain P concentrations.

Grain K concentration ranged from 030 to 0.39% in 2019 (Fig 3C) and from 0.29 to 0.43% in 2020 (Fig 4C) and decreased with plant densities 0.361, 0.353, 0.343, 0.343, 0.340, and 0.331% under 54700, 64600, 74600, 88000, 101700, and 120100 pph in 2019 and 0.374, 0.367, 0.357, 0.361, 0.349, and 0.33% under the respective plant densities in 2020. The April 30 planting showed consistently the highest grain K concentration in 2020. Grain K concentration averaged 0.338, 0.342, 0.345, 0.340, 0.350, 0.350 and 0.352% for the April 23, May 1, May 7, May 14, May 22, May 30, and June 5 plantings in 2019 and 0.380, 0.40, 0.367, 0.352, 0.352, 0.32, and 0.325% for the April 21, April 30, May 7, May 18, May 27, June 3, and June 10 plantings in 2020. Therefore, grain K concentration consistently decreased with increasing plant densities during both growing seasons; grain K concentration showed an overall increasing trend with delayed plantings in 2019 while a decreasing trend was observed in 2020.

### 3.3 Nutrient removal of maize as function of plant density and planting date

**Fig 5:**
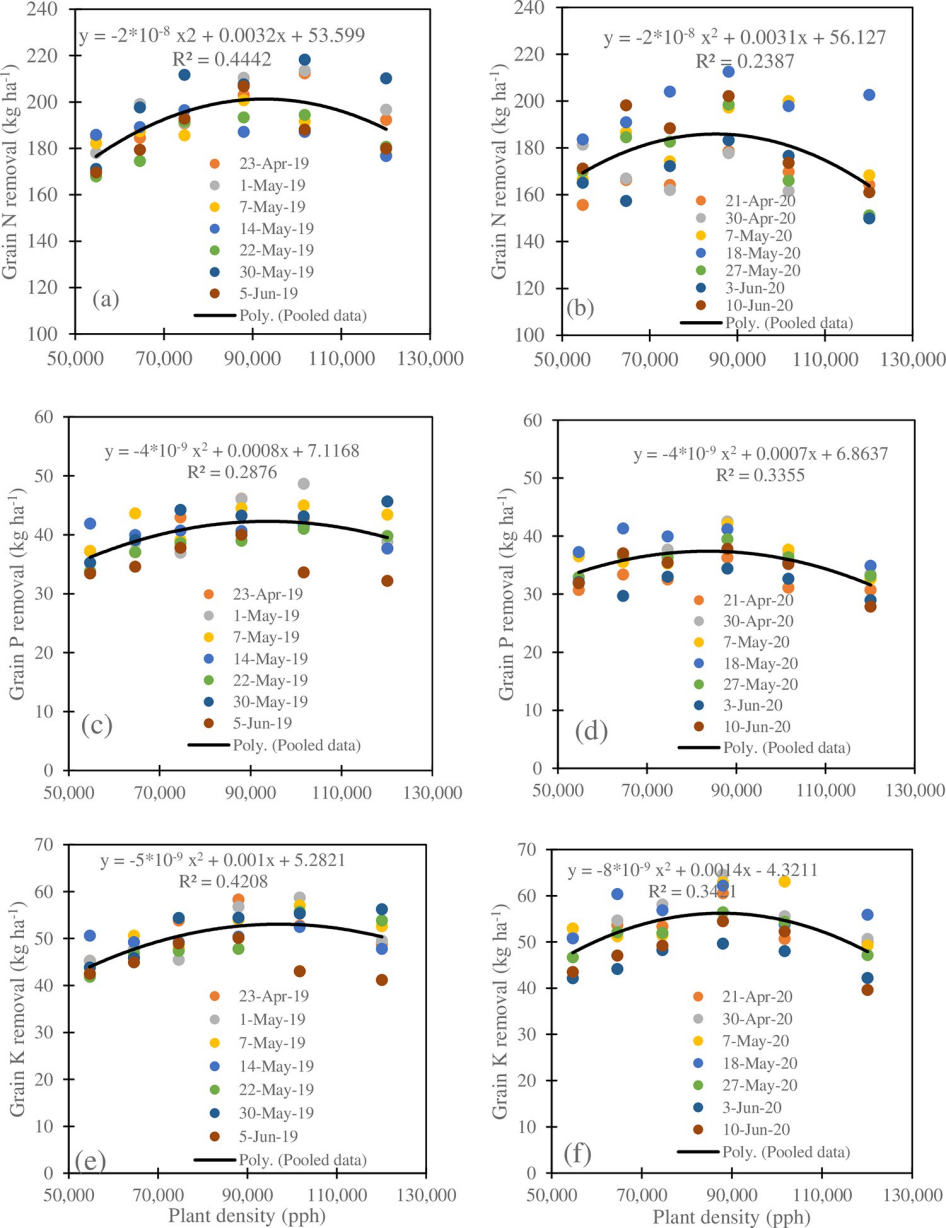
Relationships between maize grain nitrogen (a) and (b), phosphorus (c) and (d) and potassium (e) and (f) removal and the plant density during the 2019 and 2020 growing seasons.

**Fig 6:**
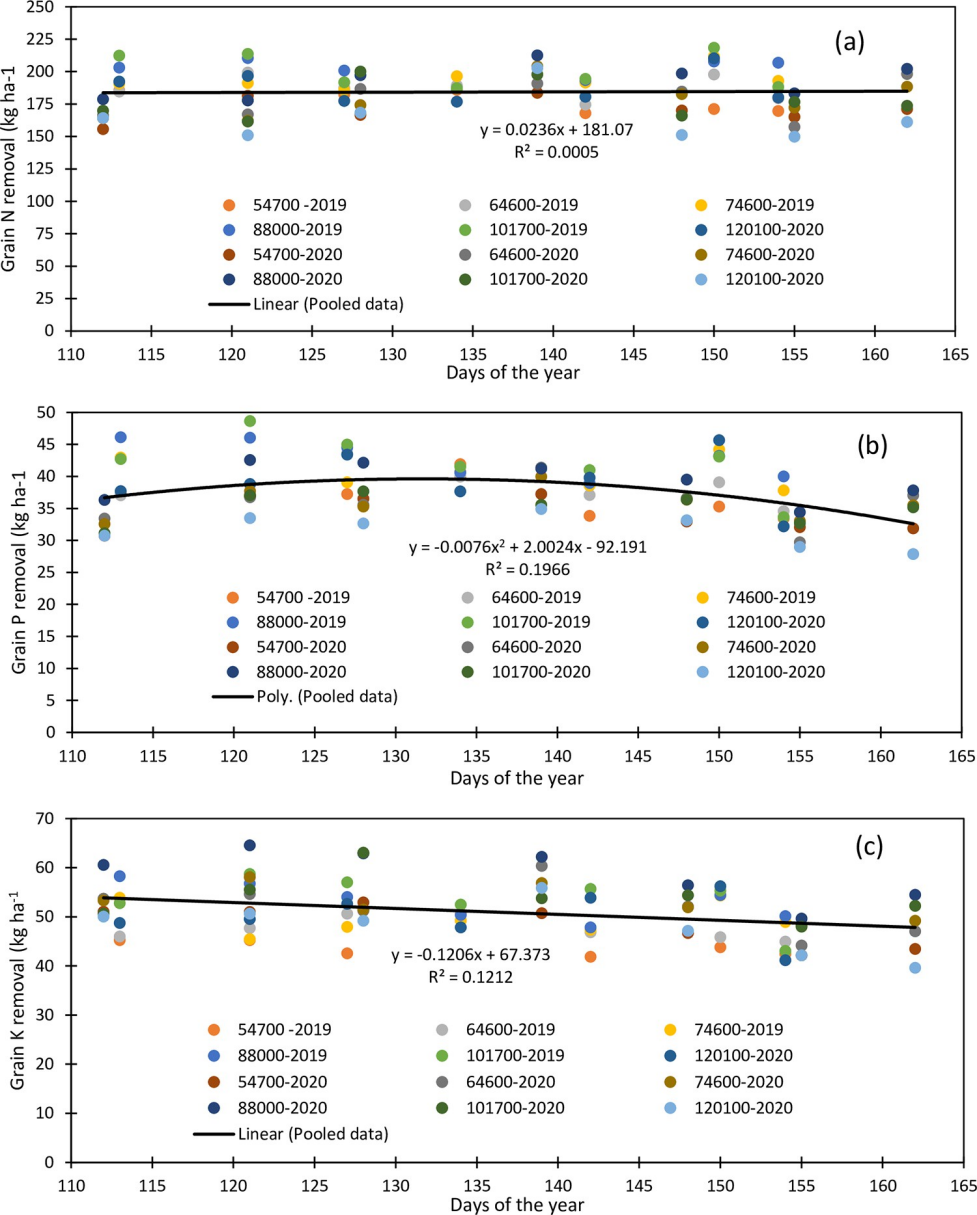
Relationships between maize grain nitrogen (a), phosphorus (b), and potassium (c) removal and the planting date (day of year) for the 2019–2020 period.

The relationship between grain N removed and plant density under different planting dates in 2019 and 2020 is presented in Fig 5A and 5B, respectively. The results indicated a polynomial second-order relationship with a higher coefficient of determination of 0.44 in 2019 than 0.24 in 2020. The amount of N removed varied from 167.94 to 218.35 kg ha^-1^ in 2019 (Fig 5A), while it varied from 149.94 to 212.57 kg ha^-1^ in 2020 (Fig 5B). Nitrogen removed averaged 177.21, 187.48, 194.35, 201.40, 200.85, and 187.75 kg ha^-1^ under 54700, 64600, 74600, 88000, 101700, and 120100 pph in 2019. It averaged 170.51, 178.71, 178.29, 192.88, 177.96, and 164.06 kg ha^-1^ under the respective plant densities in 2020. Nitrogen removal varied with the planting date. It increased from April 23 planting (194.81 kg ha^-1^) to May 1 planting (198.22 kg ha^-1^) and decreased thereafter to May 22 planting (183.79 kg ha^-1^). It rose again to May 30 planting (202.85 kg ha^-1^) to finally end up decreasing towards June 5 (186.18 kg ha^-1^) in 2019. In 2020, nitrogen removed increased from April 21 planting (166.47 kg ha^-1^) to May 18 planting (198.61 kg ha^-1^), decreased to June 3 planting (167.45 kg ha^-1^), and increased again to June 10 (182.44 kg ha^-1^) in 2020. In both years, the maximum N removal was obtained under 88,000 pph. The May 30 and May 18 plantings have the highest N removed amounts in 2019 and 2020, respectively. Grain N removal showed a very weak linear relationship (R^2^ = 0.0005) with the planting date used as days of year (DOY) with a slope of 0.0236 kg N ha^-1^ (Fig 6A). In other words, grain maize N removal increased by 23.6 g ha^-1^ with unit day planting delay.

Like grain N removed, Fig 5C and 5D show a second-order polynomial relationship between the grain P removed and plant density under various planting dates with coefficient of determination values of 0.29 and 0.34 in 2019 and 2020, respectively. The amount of P removed ranged from 32.18 to 48.66 kg ha^-1^ in 2019 and from 27.88 to 42.56 kg ha^-1^ in 2020. The P removed followed the same trend in both years and averaged 36.59, 38.59, 40.05, 42.79, 42.23, and 39.32 kg ha^-1^under 54700, 64600, 74600, 88000, 101700, and 120100 pph, respectively, in 2019 and 34.05, 35.77, 35.77, 39.15, 35.10, and 31.69 kg ha^-1^ in 2020 under the respective plant densities. The planting density of 88000 pph had the maximum P removed in both years. The planting date also impacted the amount of P removed. The results indicated that in 2019, the lowest and highest removed amount was obtained on June 5 and May 7 plantings respectively, while in 2020, the lowest and the highest removed amount was obtained on June 3 and May 18 plantings respectively. Early planting resulted in an important amount of P removed. Grain P removal showed a second order polynomial relationship (R^2^ = 0.197) with the planting date (DOY) (Fig 6B). Grain P removal increased with planting date up to the DOY 132 and decreased with the delayed planting date (Fig 6B).

The removed K amount varied with plant density and planting date and ranged from 41.18 to 58.73 kg ha^-1^ in 2019 and from 39.63 to 64.53 kg ha^-1^in 2020. Grain K removal showed a second-order polynomial relationship with the plant density with coefficient of determination R^2^ values of 0.42 and 0.34 in 2019 and 2020, respectively, (Fig 5E and 5F). Like maize grain N removal, grain K removal shower higher coefficient of determination with plant density during the first year of maize cropping than the second maize cropping year. The removed K amount increased with plant densities to a maximum value and decreased thereafter in both years with different planting density. It averaged 44.55, 47.31, 49.60, 53.12, 53.58, and 50.00 kg ha^-1^ under 54700, 64600, 74600, 88000, 101700, and 120100 pph, respectively, in 2019 and 48.29, 51.88, 52.73, 58.68, 53.94, and 47.81 kg ha^-1^under the respective plant densities. The early and late planting showed a consistently low removed K in both years, while 101700 and 88000 pph had the highest K removed value in 2019 and 2020 respectively. The amount of K removed averaged 50.82, 50.58, 50.80, 49.96, 48.91, 51.67, and 45.12 kg ha^-1^for the April 23, May 1, May 7, May 14, May 22, May 30, and June 5 plantings, respectively. In 2020, it averaged 53.21, 55.73, 55.13, 56.63, 51.44, 45.72, and 47.68 kg ha^-1^for the April 21, April 30, May 7, May 18, May 27, June 3, and June 10 plantings, respectively. Thus, a low amount of K removed is obtained in late planting, while the maximum amount is obtained on May 30 and May 18 planting in 2019 and 202 respectively. Overall, grain K removal linearly decreased with the planting date with a regression slope of -0.1206 kg K ha^-1^ and R^2^ of 0.12 (Fig 6C).

### 3.4 Soil residual nitrogen as function of plant density and planting date

The results of the analysis of variance and means pair comparison are summarized in Tables 3–5. In 2019, maize planting date and the soil sampling depth had a highly significant effect on soil residual N, P and K while the planting date only impacted soil residual K. The interaction of planting date*planting density and Planting density*sampling depth showed significant effect on residual K only. However, the interaction planting date*soil sampling depths significantly impacted residual soil N, P, and K. In 2020, the planting date and the soil sampling showed significant effect on residual soil N, P and K while the planting density had non-significant effect on soil residual N, P and K. After two continuous maize production seasons, soil residual N was the highest under D5 planting and the lowest under D1, D3, D4 and D6 plantings while soil residual P was the highest under D2, D3 and D4 plantings and the lowest under D1, D5, D6, and D7 plantings (Table 4). Soil residual K was the highest under the last planting D7 and the lowest under the first planting. The planting densities 74,600, 101,700 and 120,100 pph depleted soil of N the least and the plant densities of 54.700 and 88,000 pph depleted soil N the most. During the two growing seasons, there were no significant differences in the soil residual P regarding the plant densities. All plant densities except the 54,7000 pph showed similar soil K depletion (Table 4). Soil N, P, and K were significantly impacted by the interaction planting date and soil sampling depth and the interaction Planting date and soil sampling depth significantly impact soil N and K. The interaction planting density*soil sampling depth had no significant effect on soil residual N, P, and K. After the first year for maize production, soil N was significantly depleted in the topsoil and the middle layer more the 60–90 cm soil layer (Table 5) while after two years of maize production there was a significant downward trend in soil residual nitrogen contents. Similar trend was observed in soil residual P and K at the end of the second year of maize production regardless of planting date and planting density (Table 5).

**Table 3:** Summary of the analysis of variance (ANOVA) of the effect of maize planting date and density on the residual soil NPK.

Year	Source	df	Nitrogen	Phosphorus	Potassium	Comments
			p-value	p-value	p-value	
2019	**Main Effects**					
	Planting Date	6	0.0000	0.0442	0.0000	Significant effect on soil residual N, P and K
	Planting Density	5	0.0759	0.9795	0.0026	Significant effect only on soil residual K
	Soil Depth	2	0.0000	0.0000	0.0000	Significant effect on soil residual N, P and K
	**Interactions**					
	Planting Date * Planting Density	30	0.0899	0.1471	0.0426	Significant effect only on residual soil K
	Planting Date * Soil Depth	12	0.0332	0.0135	0.0000	Significant effect on residual soil N, P, and K
	Planting Density * Soil Depth	9	0.7942	0.8127	0.0043	Significant effect only on soil residual K
	**Errors**	60				
2020	**Main Effects**					
	Planting Date	6	0.0000	0.0008	0.0000	Significant effect on soil residual N, P and K
	Planting Density	5	0.1260	0.5427	0.1572	Non-significant effect on soil residual N, P, and K
	Soil Depth	2	0.0000	0.0000	0.0000	Significant effect on soil residual N, P and K
	**Interactions**					
	Planting Date * Planting Density	30	0.3855	0.1581	0.6608	Significant effect on residual soil N, P, and K
	Planting Date * Soil Depth	12	0.0158	0.5553	0.0000	Significant effect on residual soil N and
	Planting Density * Soil Depth	9	0.8975	0.5059	0.4424	Non-significant effect on soil residual N, P, and K
	**Errors**	60				

**Table 4:** Summary of the pair comparison of the effects of planting density and planting date on soil residual NPK at the end of the 2019 and 2020 growing seasons.

Planting date									
Year	Soil residual nitrogen			Soil residual phosphorus			Soil residual potassium		
	Planting Date	N content	Significance	Planting Date	P content	Significance	Planting Date	K content	Significance
2019	D7	17.17	a	D4	6.67	a	D7	125.39	a
	D6	10.73	b	D6	6.49	a	D3	124.11	a
	D1	6.74	bc	D7	6.22	a	D2	118.33	a
	D2	5.73	c	D2	6.19	a	D6	117.22	a
	D4	5.42	c	D3	6.19	a	D4	104.72	b
	D5	4.57	cd	D5	5.68	ab	D5	96.28	b
	D3	0.98	d	D1	4.86	b	D1	96.17	b
2020	D5	4.78	a	D3	7.23	a	D7	118	a
	D2	3.31	b	D2	6.81	ab	D6	103.11	b
	D7	3.1	b	D4	6.57	abc	D2	98.94	bc
	D6	2.46	bc	D1	5.83	bcd	D3	94.5	c
	D4	2.34	bc	D5	5.53	cd	D5	92.33	c
	D1	2.34	bc	D7	5.18	d	D4	92.22	c
	D3	2.06	c	D6	4.97	d	D1	81.89	d
Planting density									
Year	Soil residual nitrogen			Soil residual phosphorus			Soil residual potassium		
	Planting Date	N content	Significance	Planting Date	P content	Significance	Planting Date	K content	Significance
2019	101,700	9.95	a	54,700	6.2	a	101,700	121.1	a
	64,700	9.01	a	74,600	6.18	a	64,600	116.05	ab
	120,100	8.05	ab	101,700	6.1	a	74,600	113.05	abc
	74,600	6.82	ab	88,000	6.03	a	120,100	111.67	bcd
	54,700	6.04	ab	64,600	5.88	a	88,000	104.81	cd
	88,000	4.15	b	120,100	5.87	a	54,700	103.81	d
2020	120,100	3.64	a	54,700	6.53	a	64,600	101.24	a
	74,600	3.05	ab	101,700	6.19	a	101,700	101.1	a
	101,700	3.02	ab	74,600	6.16	a	74,600	98.19	ab
	64,600	2.78	ab	88,000	5.82	a	88,000	95.48	ab
	88,000	2.6	b	64,600	5.81	a	120,100	94.38	ab
	54,700	2.39	b	120,100	5.58	a	54,700	93.33	b

**Table 5:** Summary of the comparison of Soil residual NPK as impacted by the combined effect of planting density and planting date on soil sampling depth at the end of the 2019 and 2020 growing seasons.

Year	Soil residual nitrogen			Soil residual phosphorus			Soil residual potassium		
	Soil depth	N content	Significance	Soil depth	P content	Significance	Soil depth	K content	Significance
2019	60–90	11.43	a	0–30	9.5	a	0–30	166	a
	30–60	6.36	b	60–90	4.32	b	30–60	89.79	b
	0–30	4.21	b	30–60	4.31	b	60–90	79.45	c
2020	0–30	4.3	a	0–30	7.51	a	0–30	147.43	a
	30–60	2.57	b	60–90	5.68	b	30–60	75.05	b
	60–90	1.87	c	30–60	4.85	c	60–90	69.38	c

**Fig 7:**
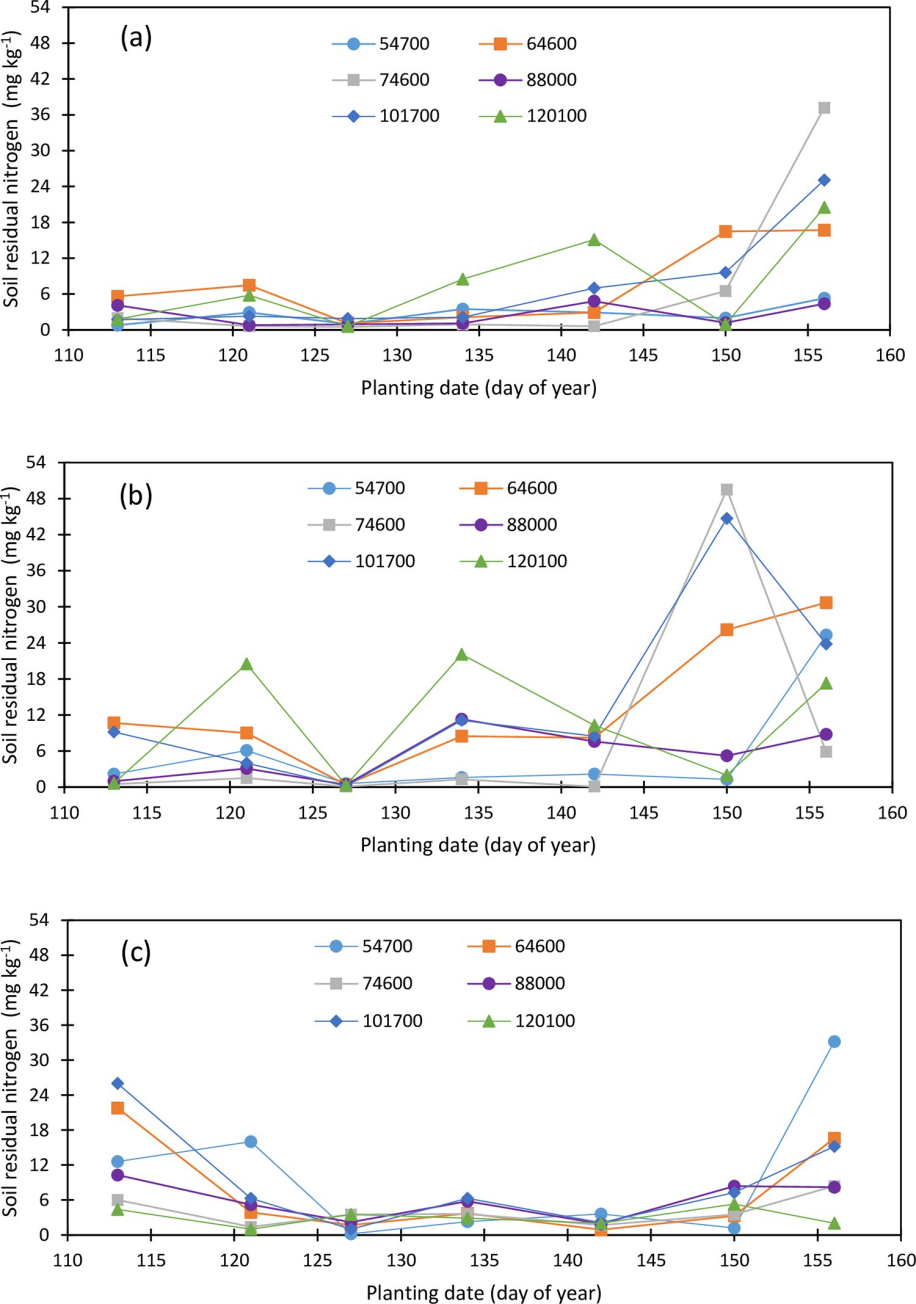
Variation in soil residual nitrogen in the (a) upper foot soil layer, (b) second foot soil layer, and (c) third soil layer as function of planting date and plant density (2019).

**Fig 8:**
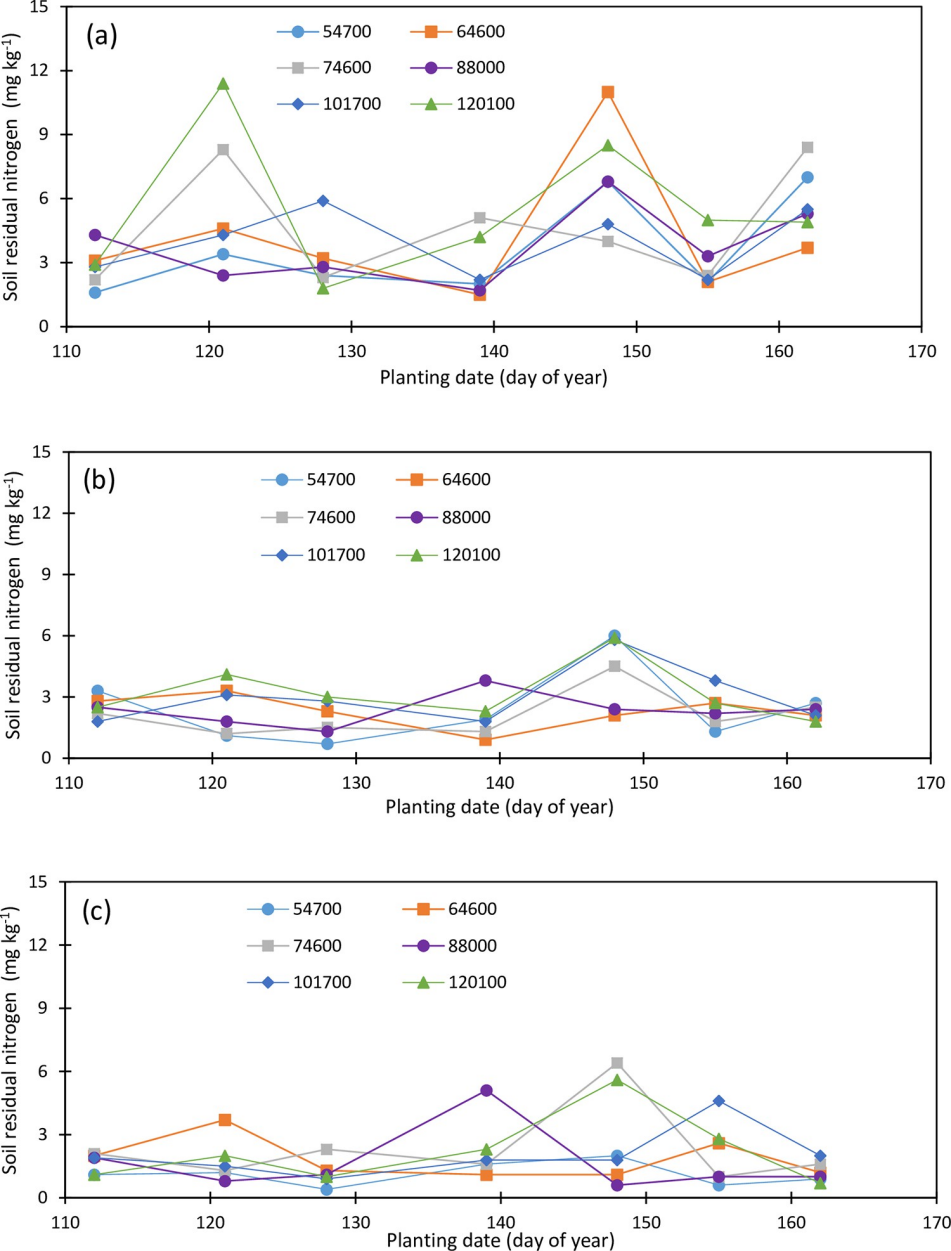
Variation in soil residual nitrogen in the (a) upper foot soil layer, (b) second foot soil layer, and (c) third soil layer as function of planting date and plant density (2020).

Soil residual nutrients varied with depth, planting density, planting date, and the crop growing season. Residual N concentration in topsoil layer 0–30 cm varied from 0.6 to 37.2 mg kg^-1^ in 2019 (Fig 7A) and from 1.5 to 11.2 mg kg^-1^ (Fig 8A) in 2020 showing the reduction in the topsoil layer from the first season to the second season. Soil N was high under the last two planting dates and averaged 2.67, 3.32, 1.0, 3.3, 5.55, 6.12, and 18.20 mg kg^-1^ under the April 23, May 1, May 7, May 14, May 22, May 30, and June 5 plantings. No trend was observed in 2020 and the topsoil N concentration averaged 2.82. 5.73, 3.07, 2.78, 6.98, 2.85, and 5.8 mg kg^-1^ under the respective planting dates. Maize plants have uptaken N from the topsoil layer the most for the May 7 planting in 2019 and April 21 planting in 2020. Topsoil N concentration averaged 2.73, 7.49. 2.90, 2.47, 7.10, and 7.60 mg kg^-1^ under 54700, 64600, 74600, 88000, 101700, and 120100 pph in 2019 and 3.61, 4.17, 4.67, 3.80, 3.96, and 5.53 mg kg^-1^ under the respective plantings in 2020.

Residual N concentration in the second soil layer was higher than N concentration in the topsoil, and it varied from 0.3 to 49.5 mg kg^-1^ in 2019 (Fig 7B) and from 0.7 to 6.0 mg kg^-1^ in 2020 (Fig 8B). It averaged 4.05, 7.37, 0.33, 9.31, 6.16, 21.48, and 18.63 mg kg^-1^ under the April 23, May 1, May 7, May 14, May 22, May 30, and June 5 plantings in 2019 and 2.52, 2.43, 1.93, 2.00, 4.45, 2.42, and 2.26 mg kg^-1^ for the April 21, April 30, May 7, May 18, May 27, June 3, and June 10 plantings in 2020 while the average soil N concentration averaged 5.61, 13.39, 8.41, 5.34, 14.50, and 10.46 mg kg^-1^ for the 54700, 64600, 74600, 88000, 101700, and 120100 pph densities in 2019, respectively, and 2.43, 2.31, 2.14, 2.34, 3.03, and 3.19 mg kg^-1^ under the respective plant densities in 2020. The second soil layer was depleted of N at the end of the second maize cropping.

The May 7, May 14, and May 22 plantings in 2019 depleted the third soil layer the most compared to the first two and the last two planting dates. The soil N concentration varied from 0.1 to 33.2 mg kg^-1^ in 2019 (Fig 7C) and from 0.4 to 6.4 mg kg^-1^ in 2020 (Fig 8C) and averaged 13.51, 5.63, 2.02, 4.10, 2.03, 4.83, and 13.93 mg kg^-1^ under the April 23, May 1, May 7, May 14, May 22, May 30, and June 5 plantings. Plant density had impacted the magnitude of the residual soil N within the third soil layer and averaged 9.87, 7.41, 4.01, 6.00, 9.17, and 3.01 mg kg^-1^ for the 54700, 64600, 74600, 88000, 101700, and 120100 pph in 2019 and 1.11, 1.86, 2.33, 1.64, 2.07, and 2.31 mg kg^-1^ in 2020 under the respective plant densities.

Combining all three soil layers, average soil N concentrations were 6.04, 9.43, 6.44, 4.60, 10.26, and 7.02 mg kg-1 under the plant densities 54700, 64600, 74600, 88000, 101700, and 120100 pph, respectively, and there were 6.74, 5.44, 1.12, 5.48, 4.58, 10.80 and 16.91 mg kg^-1^ under the first through the last planting. Soil residual N was the least under the third planting while it was the highest under the last planting. Under the delayed last two plantings, maize plants did not have much time to effectively uptake the nutrient while the third planting seemed to be the most effective planting date for maximum nutrient uptake. There was a 60% reduction in soil profile N in 2020 compared to 2019. Soil N decreased by 25, 73, and 72% in the top, mid and deep layers in 2020 compared to 2019.

### 3.5 Soil residual phosphorus as function of plant density and planting date

**Fig 9:**
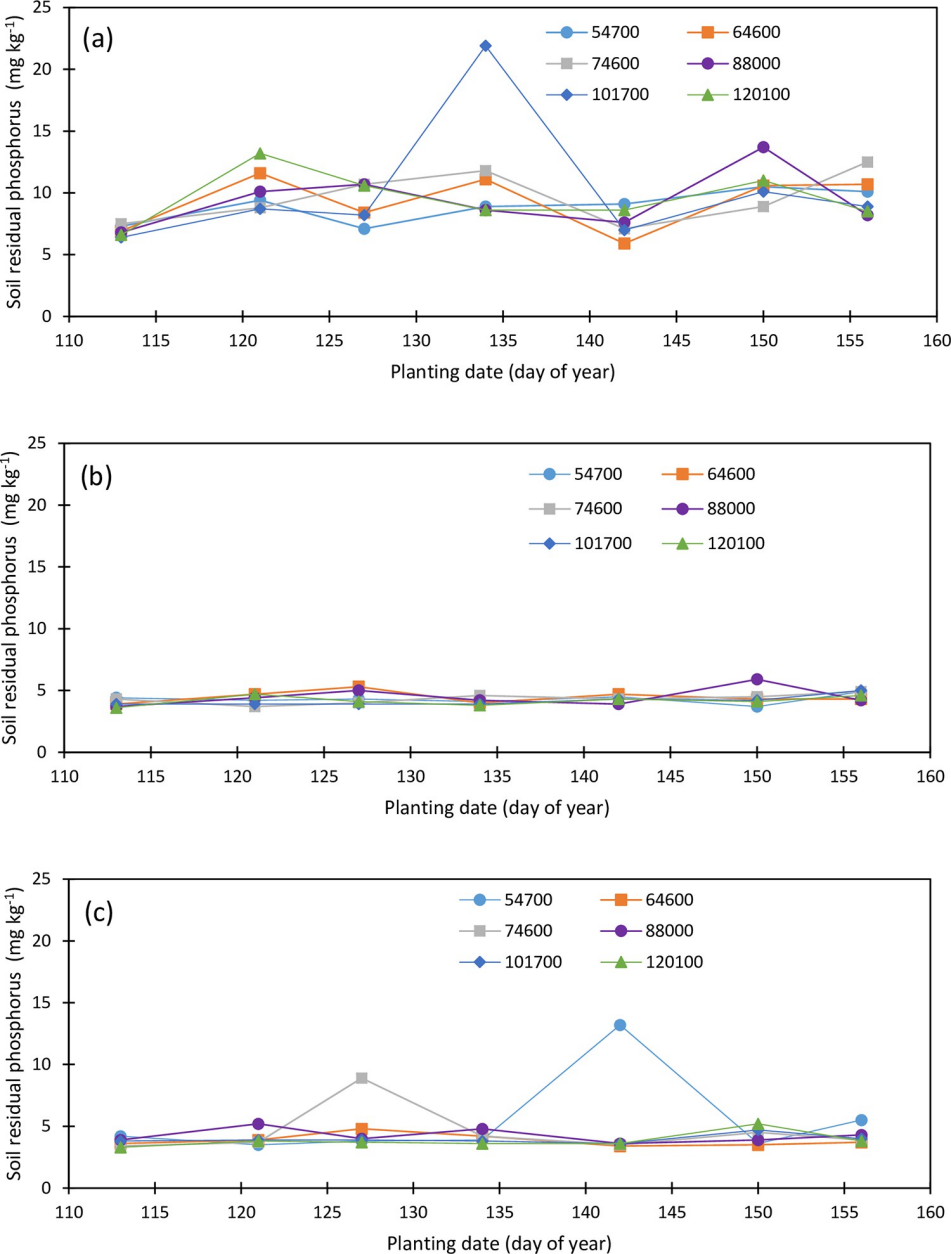
Variation in soil residual phosphorus in the (a) upper foot soil layer, (b) second foot soil layer, and (c) third soil layer as function of planting date and plant density (2019).

**Fig 10:**
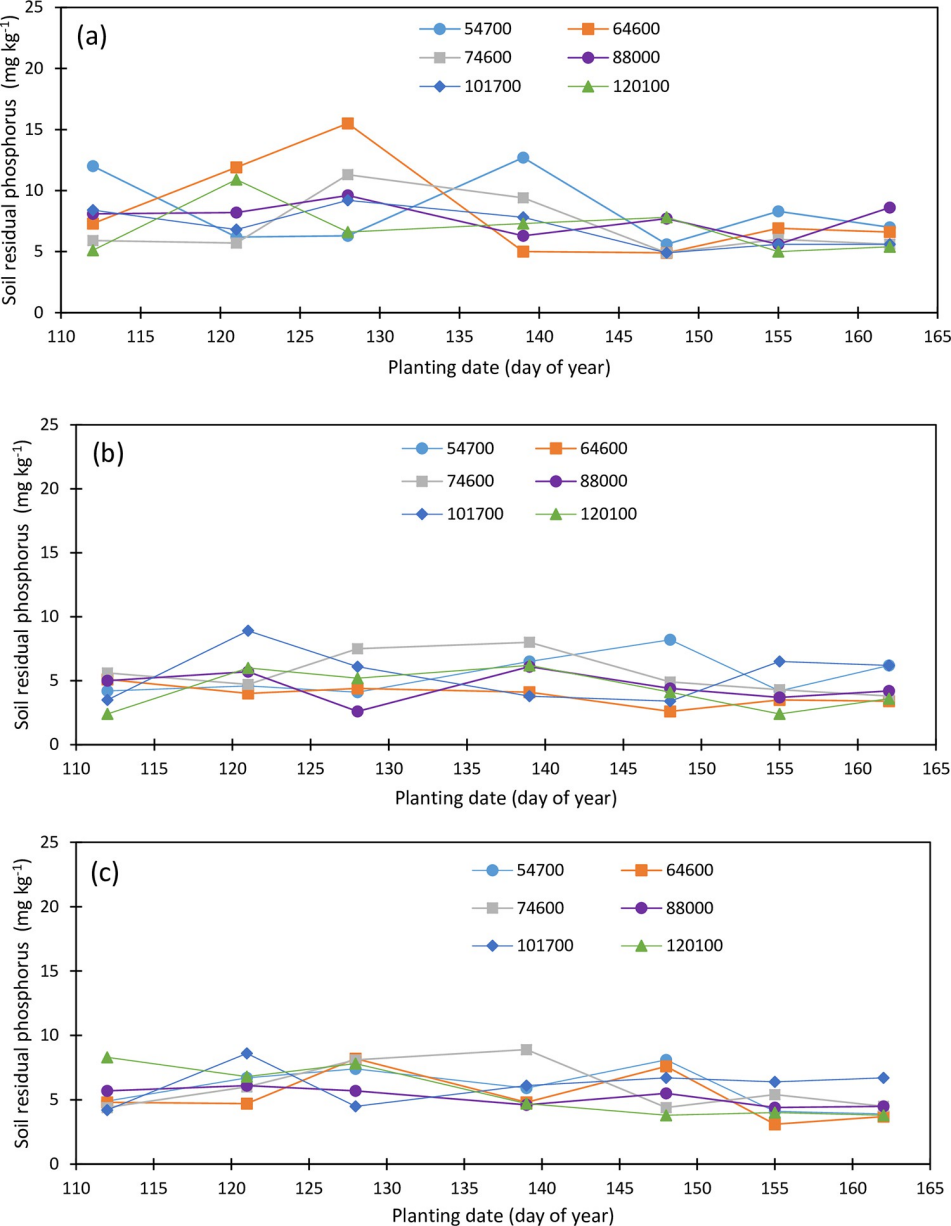
Variation in soil residual phosphorus in the (a) upper foot soil layer, (b) second foot soil layer, and (c) third soil layer as function of planting date and plant density (2020).

Initial soil phosphorus concentration was 10.6, 8.9, and 7.3 mg kg^-1^ within the topsoil, the second, and the third layers, respectively. There was a reduction in the topsoil P concentrations under different plant densities and planting dates while soil P concentrations increased within the second and third soil layers from 2019 to 2020 growing seasons. The Topsoil P concentration ranged from 5.9 to 13.7 mg kg^-1^ within an isolated point of 21.9 mg kg^-1^ for the May 14 planting under plant density 101,700 pph in 2019 (Fig 9A), and from 5.0 to 15.5 mg kg^-1^ in 2020 (Fig 10A). Soil P did not greatly vary among planting dates and densities, however, soil P exhibited a slightly increasing trend with the delayed planting and it averaged 6.92, 10.30, 9.238, 11.82, 7.55, 10.80, and 9.82 mg kg^-1^ for the April 23, May 1, May 7, May 14, May 22, May 30, and June 5 plantings, respectively, while it averaged 8.91, 9.31, 9.61, 9.39, 10.17, and 9.59 mg kg^-1^ under the 54700, 64600, 74600, 88000, 101700, and 120100 pph, respectively, in 2019. In 2020, topsoil P concentration increased from the first to the third planting and decreased under the fourth and the fifth planting dates and averaged 7.80, 8.28, 9.75, 8.08, 5.97, 6.23, and 6.47 mg kg^-1^ for the April 21, April 30, May 7, May 18, May 27, June 3, and June 10 plantings, respectively, and 8.30, 8.30, 6.97, 7.73, 6.90, and 6.87 mg kg^-1^ under the 54700, 64600, 74600, 88000, 101700, and 120100 pph, respectively.

The second and the third soil layers’ P concentration was not impacted by the planting date and plant density during the 2019 and 2020 growing seasons. The second soil layer P averaged 3.967, 4.27, 4.43, 4.10, 4.33, 4.45, and 4, 65 mg kg^-1^ for the April 23, May 1, May 7, May 14, May 22, May 30, and June 5 plantings in 2019, respectively (Fig 9B), and 4.30, 5.65, 4.98, 5.78. 4.60, 4.10, and 4.57 mg kg^-1^ for the April 21, April 30, May 7, May 18, May 27, June 3, and June 10n plantings in 2020, respectively (Fig 10B). Under the plant densities of 54700, 64600, 74600, 88000, 101700, and 120100 pph, averaged soil P concentration was 4.30, 4.46, 4.23, 4.47, 4.16, and 4.17 mg kg^-1^, respectively, in 2019 and 5.43, 3.87, 5.54, 4.53, 5.49, and 4.27 mg kg^-1^ under the respective plant densities in 2020.

Third soil layer P concentration varied from 3.3 to 13.2 mg kg^-1^ in 2019 (Fig 9C) and from 3.2 to 8.9 mg kg^-1^ in 2020 (Fig 10C). A slight decreasing trend in the third soil P concentrations with the plant densities was observed in the third soil layer in 2019. Soil P concentration averaged 5.39, 3,87, 4.59, 4.24, 3.96, and 3.85 mg kg^-1^ in 2019 and 5.56, 5.27, 5.96, 5.21, 6.17, and 5.60 mg kg^-1^ in 2020 under 54700, 64600, 74600, 88000, 101700, and 120100 pph, respectively. Soil P concentration was the lowest for the first planting in 2019 while it was the lowest for the last two plantings in 2020 and averaged 3.70, 4.00, 4.85, 4.08, 5.15, 4.23, and 4.20 mg kg^-1^ under the April 23, May 1, May 7, May 14, May 22, May 30, and June 5 plantings in 2019, and 5.38, 6.48, 6.95, 5.83, 6.02, 4.57, and 4.52 mg kg^-1^ under the April 21, April 30, May 7, May 18, May 27, June 3, and June 10 planting in 2020, respectively.

The soil profile residual P concentration averaged 6.20, 5.55, 6.18, 6.03, 6.10, and 5.87 mg kg^-1^ in 2019, and 6.53, 5.81, 6.15, 5.82, 6.19, and 5.58 mg kg^-1^ in 2020, under the plant densities 54700, 64600, 74600, 88000, 101700, and 120100 pph, respectively while it averaged 4.86, 6.19, 6.19, 6.67, 5.68, 6.49, and 6.22 mg kg^-1^ in 2019 for the April 23, May 1, May 7, May 14, May 22, May 30, and June 5 plantings, respectively, and 5.83, 6.381, 7.23, 6.57, 5.53, 4.96, and 5.18 mg kg^-1^ in 2020 for the April 21, April 30, May 7, May 18, May 27, June 3, and June 10 planting dates. Soil P concentration increased about 0.33 and 0.09 mg kg^-1^ under the plant densities 54700 and 101700 pph respectively while it slightly decreased under the rest of the plant densities from 2019 to 2020 growing seasons. Soil profile P concentration increased by about 0.97, 0.62, and 1.04 mg kg^-1^ under the first, second, and third planting dates, respectively, and it decreased by 0.10, 0.15, 1.53 and 1.03 mg kg^-1^ under the fourth, fifth, sixth, and seventh planting dates from 2019 to 2020, respectively. No change was observed in root zone soil P in 2020 compared to 2019 however, soil P decreased by 21% in the topsoil while there was an increase of 13 and 31% in soil P within the second and the third soil layers, respectively.

### 3.6 Soil residual potassium as function of plant density and planting date

Soil initial potassium concentration was 273, 89, and 43 mg kg^-1^ as of April 2019 in the topsoil, second, and third soil layers, respectively. Maize planting date and plant density affect the soil K concentration across the maize root zone. Soil K concentration within the topsoil layer varied from 53 to 126 mg kg^-1^ in 2019 and from 99 to 216 mg kg^-1^ in 2020. The second soil layer K concentration varied from 71 to 244 mg kg^-1^ in 2019 and from 49 to 103 mg kg^-1^ in 2020 while the third soil layer K concentration varied from 61 to 164 mg kg^-1^ in 2019 and from 50 to 100 mg kg^-1^ in 2020.

**Fig 11:**
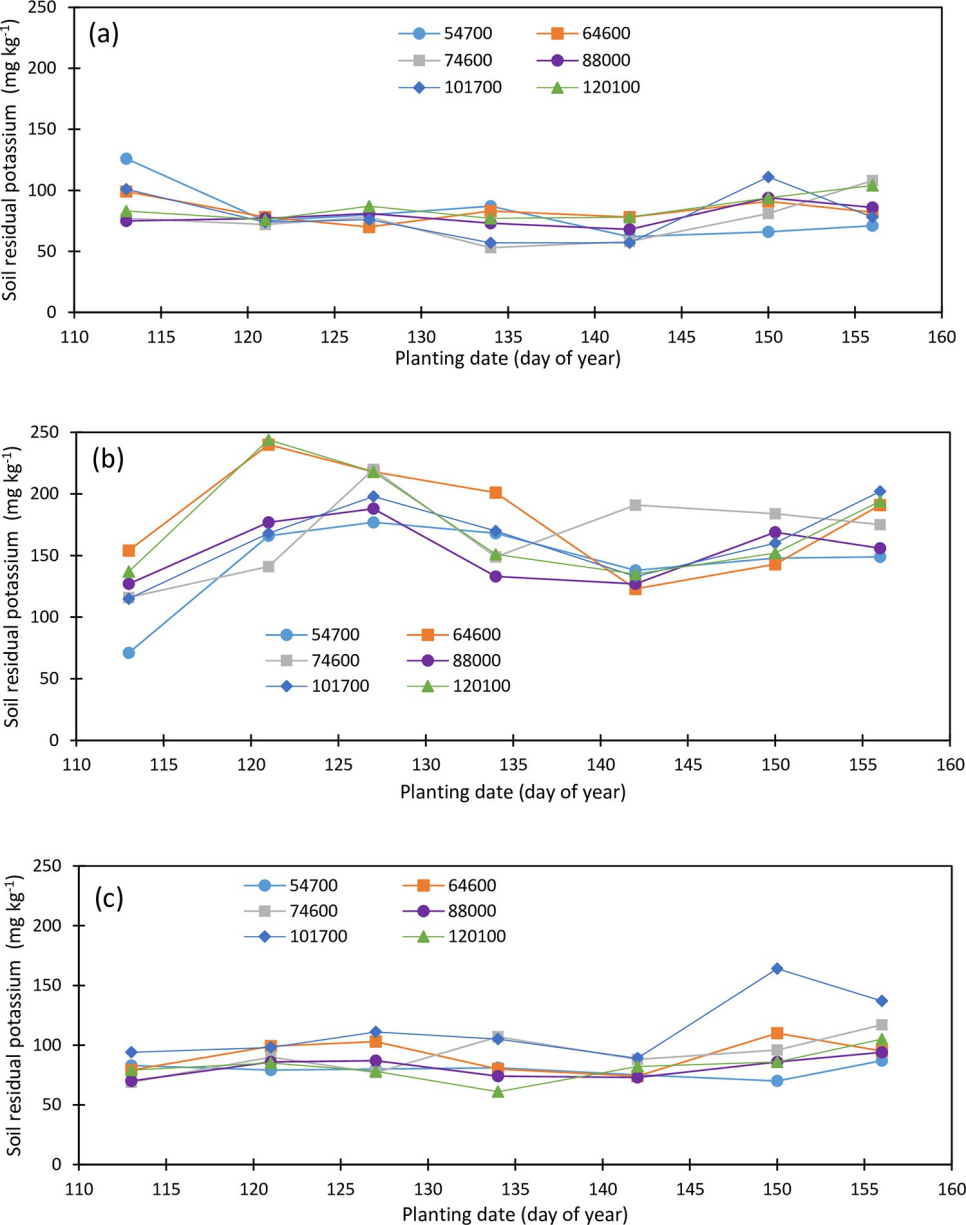
Variation in soil residual potassium in the (a) upper foot soil layer, (b) second foot soil layer, and (c) third foot soil layer as function of planting date and plant density (2019).

**Fig 12:**
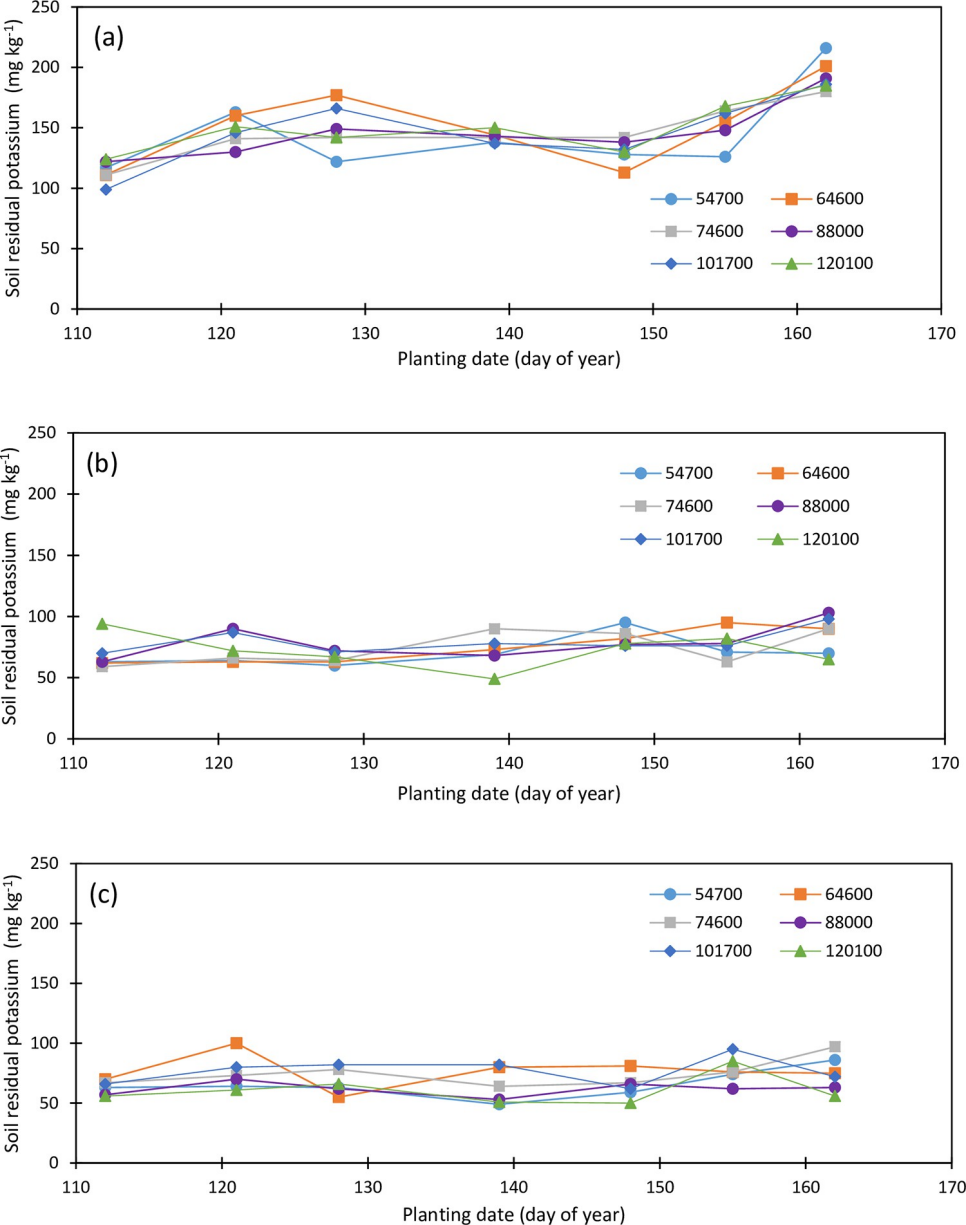
Variation in soil residual potassium in the (a) upper foot soil layer, (b) second foot soil layer, and (c) third foot soil layer as function of planting date and plant density (2020).

The topsoil K concentration is overall lower in 2019 (Fig 11A) compared to 2020 (Fig 12A) and averaged 81, 83, 75, 79, 79, and 86 mg kg^-1^ in 2019 and 144, 152, 146, 146, 147, and 150 mg kg^-1^ in 2020 for 54700, 64600, 74600, 88000, 101700, and 120100 pph, respectively, while it averaged 94, 75, 79, 72, 67, 90, and 88 mg kg^-1^ in 2019 for the April 23, May 1, May 7, May 14, May 22, May 30, and June 5 plantings, and 114, 149, 150, 142, 131, 154, and 193 mg kg^-1^ in 2020 for the April 21, April 30, May 7, May 18, May 27, June 3, and June 10 plantings, respectively. The May 14, and May 22 plantings in 2019 depleted soil K the most within the topsoil in 2019, and the April 21 and May 27 plantings in 2020 depleted the topsoil K the most in 2020. The reverse soil K concentration was observed in the second soil layer in 2020 compared to the first later in 2019. The second soil layer K averaged 145, 181, 168, 154, 164, and 176 mg kg^-1^ in 2019 (Fig 11B), and 70, 75, 74, 79, 79, and 72 mg kg^-1^ in 2020 (Fig 12B) under the 54700, 64600, 74600, 88000, 101700, and 120100 pph, respectively. The May 22 planting in 2019 is the most impacting planting date on soil residual K. Planting dates have no significant impact on soil residual K at the end of the 2020 growing season.

Soil K within the deep soil layer was also impacted by the planting density and the planting date, and there was a slight decrease in soil K (69 mg kg^-1^) in 2020 compared to 2019 (90 mg kg^-1^). Soil K concentration averaged 79, 91, 92, 81, 114, and 82 mg kg^-1^ in 2019 (Fig 11C), and 65, 77, 75, 62, 77, and 61 mg kg^-1^ in 2020 (Fig 12C) for the 54700, 64600, 74600, 88000, 101700, and 120100 pph, respectively. Soil K was higher under the last two plantings in 2019 and 2020 compared to the previous plantings and averaged 79, 90, 89, 85, 80, 102, and 106 mg kg^-1^ in 2019, and 63, 75, 68, 63, 64, 78, and 75 mg kg^-1^ in 2020. Maize plants did not have enough time to properly utilize soil K at the deeper soil depth during both growing seasons.

Maize root zone K averaged 102, 119, 112, 105, 119, and 115 mg kg^-1^ under 54700, 64600, 74600, 88000, 101700, and 120100 pph, respectively, in 2019, and 93, 101, 98, 96, 101, and 94 mg kg^-1^ in 2020 under the respective plant densities demonstrating depletion of soil K by 8.5, 17.4, 13.6, 9.3, 17.9, and 29.2 mg kg^-1^ under the respective plant densities in 2020 season compared to the 2019 season. The root zone soil K concentration averaged 98, 118, 124, 96, 117, and 124 mg kg^-1^ under the April 23, May 1, May 7, May 14, May 22, May 30, and June 5 planting dates in 2019, and 82, 99, 95, 92, 92, 103, and 118 mg kg^-1^ under the April 21, April 30, May 7, May 18, May 27, June 3, and June 10 planting dates in 2020, showing soil K depletion of 15.6, 19.1, 29.2, 13.9, 3.7, 13.8, and 5.9 mg kg^-1^ in 2020 season compared to the 2019 season from the first to the seventh planting dates. There was an 18% reduction in the rootzone soil K in 2020 compared to 2019 with disparities among soil layers. There was an 83% increase in the topsoil K in 2020 compared to 2019 and a decrease of 65 and 23% in soil K was observed in the second and third soil layers, respectively. The increase in soil K in the topsoil might be due to the 2019 biomass mineralization.

## 4. Discussion

Maize grain N, P, and K contents were significantly affected by planting date, planting density, and the planting year while only maize planting date significantly affects maize stover N, P, and K contents. Overall, grain N, P, and K showed a decreasing trend with plant density. High plant density reduces the ability of maize to use soil nitrogen due to interplant competition for solar radiation, water, and soil nutrients, and that increases crowding stress [26,51–53]. At the higher plant densities, leaf senescence occurred earlier, which probably reduces dry matter accumulation and nutrient uptake [6,40]. Raymond et al. [21] reported that nutrient uptake levels varied by planting density, with the lowest grain nutrient concentrations observed at the lowest and highest plant densities. Heckman et al. [54] reported maize grain N, P, and K concentrations of 1.29, 0.38, and 0.48%, respectively, which are higher than the finding of the present study regardless of the planting density and the planting date. The decreasing trend of grain nutrients with plant density is the dilution effect [21,55,56]. Maize grain nutrient uptake increased with plant density and decreased after 88,000 pph like the findings of Raymond et al. [21] who reported that maize grain N uptake increased with plant density up to 74,000 pph and decreased at the highest density. However, they pointed out that the lowest maize grain P concentration varied with plant density and study sites [21]. Xu et al. [57] reported grain average nitrogen removal of 163.6 and 152.2 kg ha ^-1^ while there was a significant increase in grain N removal from 152.8 to 163.0 kg ha-1 under 67,500 and 90,000 pph, respectively, at the Wuqiao Experiment Station of China Agricultural University, Hebei province, China. Soil profile may be more depleted of N under high plant density compared to low plant density [42]. Ndabamenye et al. [45] pointed out that increasing banana plant density accelerates nutrient depletion.

The results of the present study agree with Raymond et al. [21] who reported lower maize grain N removal at lower plant densities with N removal which varied with research sites and ranged from 99 to 242 kg ha^-1^. They reported variation of grain P removal with plant density and research sites, and it ranged from 21 to 53 kg ha^-1^ while grain K removal ranged from 29 to 55 kg ha^-1^. Djaman et al. [20] reported grain maize N, P, and K removal of 212, 47, and 58 kg ha^-1^, respectively under full irrigation treatment at a plant density of 73,000 pph in South-Central Nebraska. Similar results were reported by Setiyono et al. [58] and Heckman et al. [54]. Bisht et al. [59] reported higher maize N removal from 206.2 to 230.3 kg ha^-1^ under plant density varying from 66,666 to 100,000 pph while P removal ranged from 52.4 to 56.2 kg ha^-1^ and K removal was very high compared to the results of this study and ranged from 192.3 to 211.3 kg ha^-1^ under the same plant densities. The second-order polynomial relationship between grain N removal and maize plant density agrees with Raymond et al. [21]. However, Raymond et al. [21] reported a linear relationship between grain P and grain K removal and plant density while second-order polynomial relationships are reported in the present study. Integrated soil-crop system management is challenging and should be considered a priority for system sustainability while increasing crop yield to cope with the increasing world human population. Chen et al. [46] reported the need to apply about 270 kg N ha^-1^ to achieve 15.2 Mg ha^-1^ of maize under high plant densities of 90,000–105,000 pph in China. For nutrient optimization, Bruulsema et al. [60] suggested the right source of nutrients at the right rate, right time, and right place. Soil nutrient uptake by crops is correlated to root biomass and spatial distribution [61], and maize root growth was inhibited at the high plant densities of 101,700, and 120,100 pph [44,62,63]. The results of the present study agree with Zhang et al. [5] who reported a reduction in total grain nitrogen uptake at a plant density of 97,500 pph compared to 52,500, 67,500, and 82,500 pph in China. Shao et al. [63] pointed out that efficient use of P is made difficult under high densities due to the low mobility of P and the compression of crop root space.

While there was no clear variation in plant nitrogen, phosphorus, and potassium concentrations as a function of plant density, the late plantings showed higher nutrient concentrations in maize plants compared to the early plantings. Tsimba et al. [64] reported that late planting triggered a source limitation leading to plant assimilate remobilization in maize. While lower N uptake in the high maize planting density was reported by Echarte et al. [28], a non-consistent nutrient content in maize grain, stover, or cob as function of planting patterns was found by Ottman and Welch [29]. The delayed plantings exposed the maize plants to artificial plant biomass and grain drying due to the frost occurring at the end of crop season under a reduced length of the growing season [65–67]. The translocation of some nutrients such as nitrogen and phosphorus to maize grain begins at the second reproductive stage (R2) [68]. Therefore, the remobilization of assimilated from plant leaves and stover to the grain was not complete before the frost and this explains the highest nutrient contents in the plant biomass mass in the last two planting dates compared to the previous planting dates [69]. These results agreed with Zhiipao et al. [70] who reported the maize leaf and stem phosphorus content was greater in the late sown crop than the timely planting, however, they indicated that maize leaf and stem contents in nitrogen and potassium at maturity were greater in the on-time planting crops than in the delayed planted crops.

The results of this study indicated that maize roots could deplete soil nutrients to very low levels as a competition among roots. Plant density is one factor that might affect soil properties mostly when no rotation is incorporated within the cropping system. At the end of the second maize cropping season, the highest plant density of 120,100 pph registered the highest soil residual N content and the lowest plant density of 54,700 pph obtained the lowest residual soil N content while the opposite situation was observed for the residual soil P content. These results are consistent with Uwah et al. [71] who found the least soil N and P contents under maize plant density of 100,000 pph compared to the plant densities of 57,142, 66,666 and 80,000 pph. According to Sun et al. [41], root competition for soil nutrient is intensified under high plant density and promotes soil microbial activities that decompose soil organic matter and mineralize N. Similarly, these results agree with Postma et al. [42] who reported more depleted of nitrogen under high plant density compared to low plant density, even to a depth of 2 m or more however the relative depletion of nitrogen is greater deeper down, in the 1–2 m zone, than in the 0–1 m zone which is opposed to the reported results in the present study. Ndabamenye et al. [45] pointed out that increasing plant density accelerates soil nutrient depletion however, the increase in maize above biomass may contribute to nutrient partially relocated to the surface soil layer as the biomass in incorporated to the soil after harvest as a common practice in the study area. Plant population should be balanced with the sum of soil nutrient and the applied nutrient as fertilizer for system sustainability. For forest plantation, Selvakshmi et al. [72] found significant impact of planting densities, soil depths, and their interaction had a significant effect on soil quality index with soil quality degradation under increasing plant density of pine (*Pinus kesiya*). Duan et al. [43] indicated that excessively high planting density is not beneficial to the long-term maintenance of soil fertility in Chinese fir (*Cunninghamia lanceolata* (Lamb.) Hook) plantations. The soil N and P contents decrease with the increase in soil depth. in five Chinese fir density stands decreased with the increase in soil depth while high-density stands are beneficial to the accumulation of soil total K [43]. Munnaf et al. [73] investigated five management zones in a field based on nine soil chemical properties and found that maize planting density should be appropriately increased under high soil fertility, and appropriately decreased under poor soil fertility. At the increasing planting density beyond the optimum, the capacity of the crop to accumulate nitrogen per unit of green LAI decreased [40]. The present study highlighted the necessity to combine plant density, and planting date to balance N, P, K fertilizers to match and synchronize crop demand and nutrient delivery for system sustainability. Additional research on incorporating variable rates of fertilizer under the studied plant densities is needed to improve maize grain yield and nutrient use efficiency (NUE). We hypothesized that under high plant densities, an increased rate of N application is necessary to meet the demand of N for maximum grain yield. As demonstrated through the two-year research, the optimum plant density for achieving maximum grain yield is impacted by the prevailing climatic conditions.

## 5. Conclusions

Maize fertilizer recommendations are provided regardless of the plant density and the planting date which may impact plant nutrient uptake and the soil chemical properties at the end of the cropping period. This research evaluated maize nutrient uptake and soil residual nutrient under six plant densities ranging from 54,700 to 120,100 plants per hectare and seven planting dates from April 20 to June 10. Crop plots were managed uniformly across different planting densities and dates during the 2019 and 2020 growing seasons. The results showed significant variation in plant N, P, and K concentrations at crop maturity under different planting dates while maize planting densities showed non-significant effect on plant N, P, and K concentrations The last two planting dates showed higher nutrient concentrations in maize plants at maturity. Maize grain N, P, and K concentrations significantly decreased with increasing plant density. Grain nutrient concentration was also significantly affected by planting date and the growing season. Significant variations in soil residual N, P, and K were observed across the soil profile under different planting dates. Soil residual N was the lowest under May 7 planting in 2019 and it was the highest for the last planting in 2019 while no trend was observed in 2020. Soil residual N decreased by 25, 73, and 72% in the 0–30 cm, 30–60 cm, and 60–90 cm, soil layers in 2020 compared to 2019. Maize plant density did not affect soil residual P, however, soil P decreased by 21% in the 0–30 cm soil layer while there was an increase of 13 and 31% in soil P within the 30–60 cm and 60–90 cm soil layers, respectively. Soil residual N, P, and K were significantly affected by the sampling depth. Topsoil residual K was low in 2019 and had significantly increased in 2020 while the 30–90 cm soil profile showed a significant decrease in soil K with no differences among maize plant densities and planting dates. There was an 83% increase in 0–30 cm soil layer K in 2020 compared to 2019 and a decrease of 65 and 23% in soil K was observed in the 30–60 cm and 60–90 cm soil layers, respectively. Soil nitrogen was depleted under the optimum planting window for different planting densities, and this calls for future perspective in investigating fertilizer management options under the optimum planting window and planting density to improve maize yield and nutrient use efficiency while maintaining a sustainable maize production system. Grain nutrient removal and soil residual nutrient profiles indicate that plant density should be adjusted with nitrogen fertilizer amount for improving maize productivity and for system profitability and sustainability in the sandy loam soil in New Mexico.

## Supporting information

S1 Data (XLSX)

S2 Data (XLSX)

S3 Data (XLSX)

S4 Data (XLSX)
